# Sitting vs. supine ultrasound measurements of the vastus medialis: correlations with MRI measurements and age considerations

**DOI:** 10.1186/s40101-023-00331-6

**Published:** 2023-07-15

**Authors:** Masashi Taniguchi, Yoshihiro Fukumoto, Masahide Yagi, Tetsuya Hirono, Momoko Yamagata, Ryusuke Nakai, Yosuke Yamada, Misaka Kimura, Noriaki Ichihashi

**Affiliations:** 1 Human Health Sciences, Graduate School of Medicine, Kyoto University, Kyoto, 606-8507 Japan; 2 Faculty of Rehabilitation, Kansai Medical University, Hirakata, 573-1136 Japan; 3 School of Health and Sport Science, Chukyo University, Aichi, 470-0393 Japan; 4 Japan Society for the Promotion of Science, Tokyo, 102-0083 Japan; 5 Kyoto University Institute for the Future of Human Society, Kyoto, 606-8507 Japan; 6 National Institute of Health and Nutrition, National Institutes of Biomedical Innovation, Health and Nutrition, Tokyo, 162-8636 Japan; 7 Institute for Active Health, Kyoto University of Advanced Science, Kyoto, 621-8555 Japan

**Keywords:** Vastus medialis, Measurement posture, Muscle thickness, Echo intensity

## Abstract

**Background:**

Muscle thickness (MT) and echo intensity (EI) measurements are ultrasound alternatives to magnetic resonance imaging (MRI) for evaluating muscle quantity and quality. The vastus medialis (VM) is a clinically important muscle, and assessment methods that most accurately reflect its quantity and quality are required. This study aimed to examine the correlation between MT and EI measured in the supine and sitting postures with corresponding MRI-measured muscle quantity and quality indices.

**Methods:**

In total, 134 adults (91 older and 43 young) participated in this study. Ultrasound images of the VM were acquired in the supine and sitting postures, and MT and EI were measured. The cross-sectional area (CSA), muscle volume (MV), and intramuscular adipose tissue (intraMAT) of the VM were evaluated from MRI images using T1-weighted and Dixon methods. Pearson’s coefficients were used to quantify the correlation strength amongst pairs of dependent variables. Meng’s test was used to test for correlation coefficient differences between the two measurement postures (supine and sitting).

**Results:**

The correlation coefficients amongst MT, CSA, and MV were significantly higher in the sitting posture than in the supine posture. EI measured in the supine and sitting postures correlated significantly with intraMAT, and in young individuals, these correlation coefficients were significantly higher in the sitting posture than in the supine posture.

**Conclusions:**

These findings suggest that assessment of VM muscle quantity in the sitting posture is superior for young and older individuals, and assessment of VM muscle quality in the sitting posture is most effective in younger individuals.

**Supplementary Information:**

The online version contains supplementary material available at 10.1186/s40101-023-00331-6.

## Introduction

Age-related muscle weakness is associated with atrophy and loss of muscle quality, such as an increase in intramuscular adipose tissue (intraMAT) infiltration [1–3]. While magnetic resonance imaging (MRI) is the gold standard for quantifying muscle volume (MV) as a quantity index and intraMAT as a quality index, it is time-consuming for acquisition and analysis. B-mode ultrasound (US) imaging is a convenient, cost-effective, and noninvasive alternative. Several previous B-mode US studies [4–8] have used muscle thickness (MT) as a muscle quantity index and muscle echo intensity (EI) as a muscle quality index. Decrease in MT indicates loss of muscle mass, while enhanced EI reflects an increase in intraMAT infiltration [9, 10]. Although there is no doubt that muscle mass is larger in men than in women, an increased intraMAT is not different between sexes [11]. Given that MT and EI are negatively correlated in older individuals [12], the loss of muscle quantity and quality is relatively more affected by ageing than by sex [11]. Thus, assessment of MT and EI may be useful in detecting age-related changes.

Many studies have focused on degeneration of the vastus medialis (VM) muscle because it has the second largest muscle cross-sectional area (CSA) in the quadriceps group and because it functions as a dynamic stabilizer of the knee [13–15]. VM assessment is commonly conducted in the supine posture [16–18] with high reproducibility [19]. However, MT and EI measurements can conceivably be influenced by changes in muscle morphology resulting from pressure on the posterior thigh from the bed surface while supine. It is also conceivable that VM morphology changes less while sitting on the edge of a chair because the pressure on the posterior distal thigh is reduced. An additional conceivable advantage of sitting is measurement ease; an examiner can easily operate the probe to identify the thickest site of the VM. While a previous study [20] confirmed that differences in measurement posture can change muscle shape, which could influence both MT and EI, to the best of our knowledge, no previous report has compared sitting and supine postures in terms of muscle quantity and quality indicators.

Provided one measures a single muscle, EI measurement results are unaffected by measurement location [21]. However, both MT and EI measurements are expected to be posture dependent because (a) changing from supine to sitting stretches the quadriceps, implying that the VM’s MT is thinner when sitting than when supine, and (b) MT changes affect echo attenuation, which in turn implies posture-dependent EI. While posture dependence of absolute MT and EI values is expected, it is unclear whether their correlations with corresponding MRI indices are also posture dependent. It is also unclear whether these potentially posture-dependent correlations are affected by ageing.

This study aimed to examine the correlation between MT and EI measured in the sitting and supine postures with their corresponding MRI indicators: CSA, MV, and intraMAT. Due to more easily identifiable measurement site when sitting, we hypothesized that MT is more strongly correlated with CSA and MV when sitting than when supine. We also hypothesized that the EI value measured in the sitting posture was higher than that in the supine posture because of the thinning of the MT, and that the correlation between EI and intraMAT in the young subgroup was stronger in the sitting posture than in the supine posture. This verification will help to establish more optimal measurement methods for MT and EI measurements of the VM using B-mode US images.

## Materials and methods

### Study participants and procedures

Ninety-one healthy older adults aged > 60 years (women, *n* = 49 [53.8%]; mean age, 75.3 ± 6.6 [range, 60–89] years) and 43 healthy young adults (women, *n* = 21 [48.8%]; mean age, 26.1 ± 4.7 [range, 20–39] years) were enrolled in this study. The inclusion criteria were as follows: independent community living, no assistive walking devices, no history of lower extremity surgery, and no general contraindications for MRI. The exclusion criteria were as follows: medical history of rheumatoid arthritis, neurological disorders including stroke and Parkinson’s disease, and cognitive dysfunction. Before initiating the examination, the purposes and procedures were explained to the participants, and they provided written informed consent. The study protocol was approved by the Ethics Committee of Kyoto University Graduate School and Faculty of Medicine (R1746).

The participants were instructed to avoid excessive physical activity prior to measurements. Upon arrival at the laboratory, participants rested in a sitting or supine posture and were allowed a small amount of water intake. Ultrasound and MRI measurements were randomly performed on the same day. Before the US and MRI acquisitions, the measurement site of the VM described below was marked on the skin.

### Ultrasound measurements

**Fig. 1:**
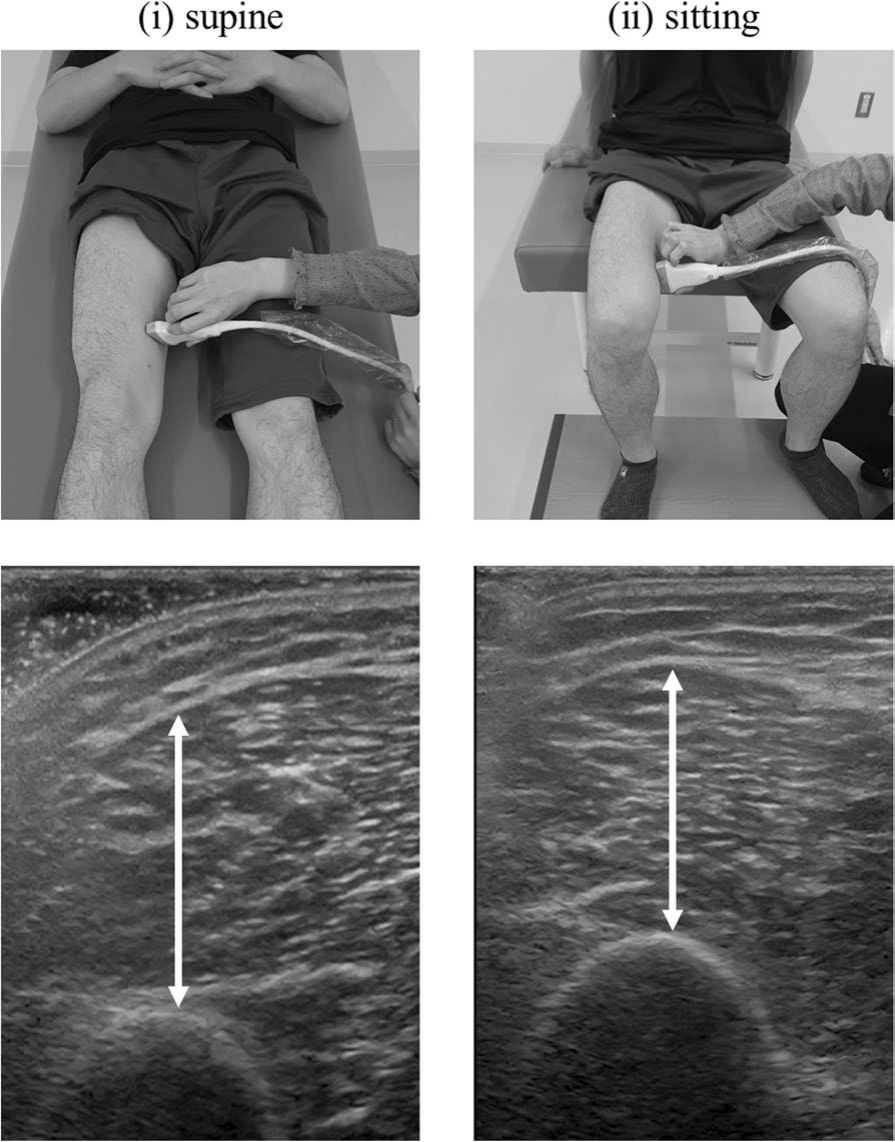
Measurement postures and representative ultrasound images of the vastus medialis captured in (i) supine and (ii) sitting postures. Ultrasound images of the vastus medialis in supine and sitting postures of young men who participated this study

**Fig. 2:**
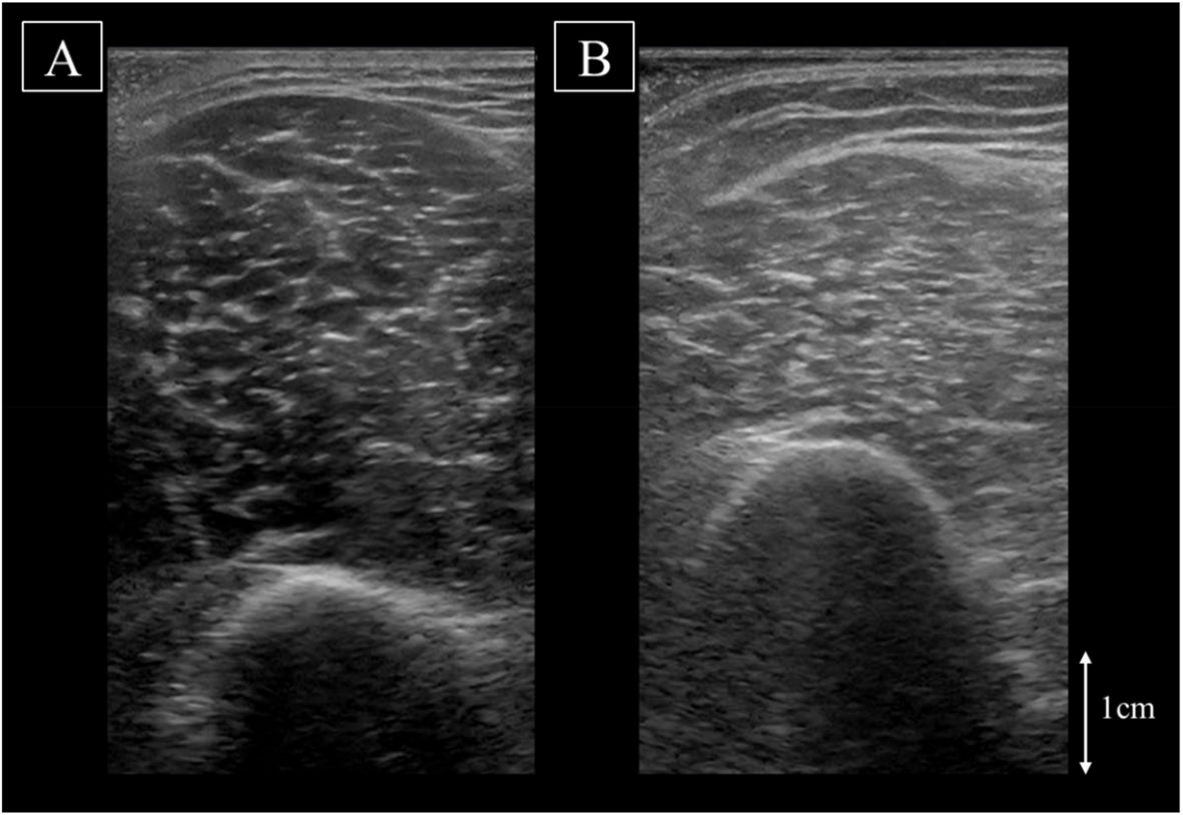
Representative ultrasound images measured in the sitting posture of **A** young and **B** older individuals. The young participant exhibits thick MT and low EI, while the older participant exhibits thin MT and high EI. The higher density of white values in the older participant reflects increased intraMAT

A real-time B-mode US device (LOGIQ e, GE Healthcare, UK) with an 8–12-MHz linear transducer was used to acquire transverse images of the VM on the right thigh. The settings for obtaining US images were unified for all measurements at a frequency of 8 MHz, dynamic range of 69 dB, and time gain of 58 dB, The measurement site was set on the VM muscle area medially displaced from the 30% distal between the greater trochanter and lateral femoral tuberosity. This VM site has the largest CSA [22]. The two scanning postures were as follows (Fig. 1): (1) supine posture (the participants laid supine and relaxed completely, and their knee joints were set in extension with neutral posture of the hip) and (2) sitting posture (the participants were seated on the edge of one’s chair to avoid contact between the posterior surfaces of the thighs and chair, with 90° of knee flexion and foot on the floor). The contralateral lower leg (hip joint) was slightly abducted as needed to facilitate transducer use. The investigator applied the transducer perpendicular to the longitudinal axis of the femur while avoiding compressive tissue deformation using a water-soluble transmission gel. Subsequently, the investigator carefully adjusted the transducer to the location of the thickest MT (i.e. the distance from the subfascia and femur). The dynamic focus depth was located at the middle of the VM to consider the effect of depth-dependent attenuation of the EI [10]. Three investigators with over 5-year experience of US operation conducted these measurements. MT was measured as the maximum distance from the muscle fascia to the femur. For EI analysis, image analysis software (ImageJ-WinJP; LISIT, Japan) was used, first to convert the data to 8-bit greyscale, yielding EI values with arbitrary units (a.u.) from 0 to 255 (black to white), where enhanced EI reflected an increase in intraMAT infiltration (Fig. 2) [9]. The region of interest (ROI) was set as the maximum-sized rectangle that encapsulated the VM without also encapsulating fascia and bone. The mean EI was quantified as the average pixel intensity within the ROI. These analyses of MT and EI were performed by an investigator other than the investigator who obtained the US images. The intraclass correlation coefficient (ICC) (1.1) values for the MT and EI in the supine and sitting postures were 0.87 and 0.98 and 0.81 and 0.93, respectively. The ICC (2.1) values for the MT and EI in the supine and sitting postures were 0.81 and 0.83 and 0.76 and 0.81, respectively.

### MRI measurements

Before starting the MRI acquisition, a surface marker was placed at the US measurement site. T1-weighted and 2-point Dixon MRIs of the right thigh from the pelvis to the right tibial tuberosity were acquired using a 3.0 T MRI scanner with a body matrix coil and a spine coil (MAGNETOM Verio; Siemens AG, Germany), as described previously [23]. The multi-slice sequence with a slice thickness of 4 mm was conducted with the following acquisition parameters: repetition time (TR), 2820 ms; echo time (TE), 16 ms; field of view, 320 × 240 mm; flip angle, 129°; and voxel size, 0.5 × 0.5 × 4.0 mm. Two-point Dixon images were obtained using the following sequences [10]: slice thickness, 3 mm; TR, 4.33 ms; TE1, 1.31 ms; TE2, 2.54 ms; optimized field of view, 286.4 × 365 mm; and flip angle, 9°. Based on the water/fat chemical shift difference and, consequently, on their phase difference in signal intensity, water and fat images were produced in- and opposed-phase sequences. IntraMAT was calculated using the following equation: intraMAT (%) = mean signal intensity of fat × 100/(mean signal intensity of fat + mean signal intensity of water). The ROIs in the VM were registered using OsiriX MD (version 11.0; OsiriX, Switzerland). To obtain the MV, the ROIs were traced on each imaging slice throughout the entire muscle length, and the muscle CSA (cm^2^) within the ROIs was measured. The volume (cm^3^) in each slice was determined by multiplying the muscle CSA by 4 mm of slice thickness, and the MV was calculated by summing the volume in all slices. In addition, CSA was calculated in the slice corresponding to the US image, as indicated by the surface marker. To calculate the intraMAT, the ROIs were carefully traced within the fascial borders. The ROIs for the VM were set in 10 consecutive slices at the US measurement site as the centre. IntraMAT (%) was determined as the average value of 10 slices [10].

### Statistical analyses

Before all statistical tests, we confirmed no outliers greater than the mean ± 3 standard deviations (SDs). The Kolmogorov–Smirnov test was conducted to assess for normally distributed data for all variables. Two-way analyses of variance (ANOVAs) with age and posture as factors were performed for MT and EI, and then the Tukey test for post hoc comparisons amongst postures was conducted. As the older and young subgroup analysis, the values of MT and EI in the supine and sitting postures were compared using paired *t*-tests. We used Pearson’s coefficients to quantify the correlation strengths amongst both (a) MT, MV, and CSA and (b) intraMAT and EI for the two measurement postures. The difference in the correlation coefficients between the two measurement postures was examined using Meng’s test [24]. For secondary analyses, these tests were conducted separately for the older and young subgroups. Furthermore, as complementary analyses, we used Spearman’s correlation coefficients to test the correlation between the change ratios in MT and EI due to the measurement posture change in each older and young subgroup. Statistical analyses were performed using the Statistical Package for the Social Sciences (version 25.0, IBM Japan Inc., Armonk, NY, USA). Statistical significance was set at *p* < 0.05.

## Results

A summary of the muscle properties measured using MRI is shown in Table 1. Amongst all participants, the CSA, MV, and intraMAT measured using MRIs were 15.1 ± 4.5 cm^2^, 301.2 ± 98.5 cm^3^, and 6.5 ± 2.1%, respectively. Result of the two-way ANOVA for MT showed no significant interaction (*F* = 0.26, *p* = 0.613) but showed a significant main effect in both age and posture (age: *F* = 30.77, *p* < 0.001; posture: *F* = 10.60, *p* = 0.001). Similarly, the results for EI showed no significant interaction (*F* = 0.51, *p* = 0.474) but showed a significant main effect in both age and posture (age: *F* = 68.02, *p* < 0.001; posture: *F* = 31.85, *p* < 0.001). The results of the Tukey test showed MT when sitting was thinner than when supine, whereas EI was higher when setting than when supine (Table 2 Total sample). In addition, the results of each young and older subgroup on MT and EI were also similar to those of total sample (Table 2 Young and older).

**Table 1:** Summary of participant’s characteristics and muscle properties measured using magnetic resonance imaging

	Pooled	Older			Young		
		Total	Men	Women	Total	Men	Women
	*n* = 134	*n* = 91	*n* =40	*n* = 51	*n* = 43	*n* = 22	*n* = 21
Age, years	59.8 ± 23.8	75.4 ± 6.7	76.7 ± 6.2	74.4 ± 7.0	26.1 ± 4.7	27.3 ± 4.4	24.9 ± 4.7
Height, cm	160.7 ± 8.9	158.5 ± 9.0	166.1 ± 6.0	152.1 ± 5.6	165.5 ± 6.6	170.4 ± 4.3	160.4 ± 4.3
Weight, kg	55.3 ± 8.7	54.2 ± 9.0	60.8 ± 7.9	48.8 ± 5.7	57.8 ± 7.7	63.0 ± 5.5	52.5 ± 5.6
Body mass index, kg/m^2^	21.4 ± 2.4	22.0 ± 2.6	21.1 ± 2.6	21.5 ± 2.6	21.0 ± 1.9	21.7 ± 1.5	20.4 ± 2.2
MV, cm^3^	301.2 ± 98.5	267.9 ± 80.5	325.0 ± 60.7	220.9 ± 62.5	373.2 ± 97.4	451.4 ± 63.7	291.3 ± 43.6
CSA, cm^2^	15.1 ± 4.5	13.8 ± 3.9	16.2 ± 3.5	11.8 ± 3.1	17.9 ± 4.4	21.1 ± 2.9	14.5 ± 2.9
IntraMAT, %	6.5 ± 2.1	7.0 ± 2.2	6.5 ± 1.5	7.5 ± 2.7	5.4 ± 1.1	5.1 ± 0.8	5.7 ± 1.4

**Table 2:** Comparisons of muscle thickness and echo intensity in the supine and sitting postures evaluated using ultrasound

	Supine	Sitting	*T*-value	Diff.	95% *CI*	*p*-value	Effect size
Tukey test	Mean ± SD						Partial *η*^2^
Total sample							
MT, cm	3.30 ± 0.64	3.04 ± 0.57	3.35	0.24	0.10 to 0.39	< 0.001	0.04
EI, a.u.	68.0 ± 15.4	78.1 ± 13.9	−5.63	−10.91	−14.72 to −7.10	< 0.001	0.11
Paired *t*-test							Cohen’s *d*
Young							
MT, cm	3.56 ± 0.53	3.35 ± 0.51	3.45	0.20	0.08 to 0.32	< 0.001	0.40
EI, a.u.	59.2 ± 11.7	67.7 ± 10.2	−5.47	−8.45	−11.57 to −5.34	< 0.001	0.77
Older							
MT, cm	3.19 ± 0.66	2.90 ± 0.54	6.24	0.28	0.19 to 0.38	< 0.001	0.48
EI, a.u.	72.1 ± 15.3	83.0 ± 12.6	−8.64	−10.91	−13.42 to −8.40	< 0.001	0.78

In total sample, results of the Tukey test for post hoc comparison against posture factor following two-way ANOVA are presented. In the older and young subgroups, results of paired *t*-test amongst MT and EI in the supine and sitting postures are presented

**Fig. 3:**
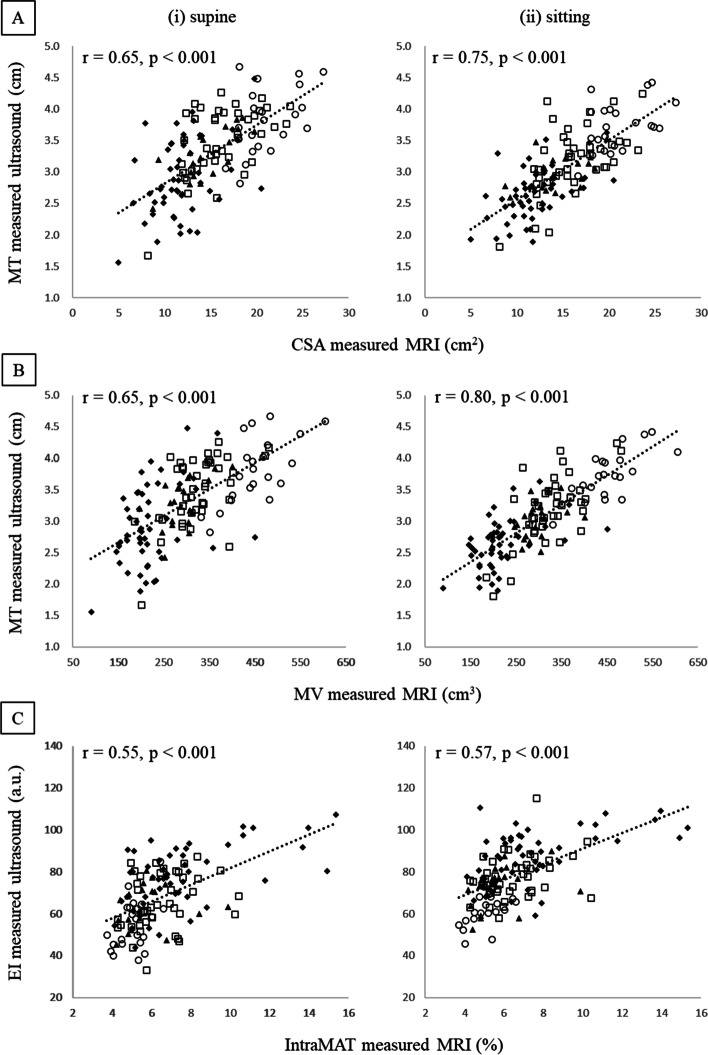
Scatter plot for correlation analysis. **A** Scatter plot of cross-sectional area and muscle thickness (MT) measured in (i) supine and (ii) sitting postures. **B** Scatter plot of muscle volume and MT measured in (i) supine and (ii) sitting postures. **C** Scatter plot of intramuscular adipose tissue and echo intensity measured in (i) supine and (ii) sitting postures. A *p*-value < 0.05 (within each scatter plot) on the Pearson correlation coefficient indicates a significant difference between parameters measured by magnetic resonance imaging and ultrasound. Each plot means older men (□), older women (◆), young men (〇), and young women (▲)

Figure 3 shows the correlations between MT and CSA and MV and that between EI and intraMAT in the supine and sitting postures. Although significant correlations between MT and CSA and MV were observed for both the supine and sitting postures (Fig. 3a, b), Meng’s test showed that the correlation coefficient in the sitting posture was significantly higher than that in the supine posture. In contrast, intraMAT and EI in the supine and sitting postures showed significant correlations (Fig. 3c), and there was no significant difference in the correlation coefficient between the two measurement postures.

**Fig. 4:**
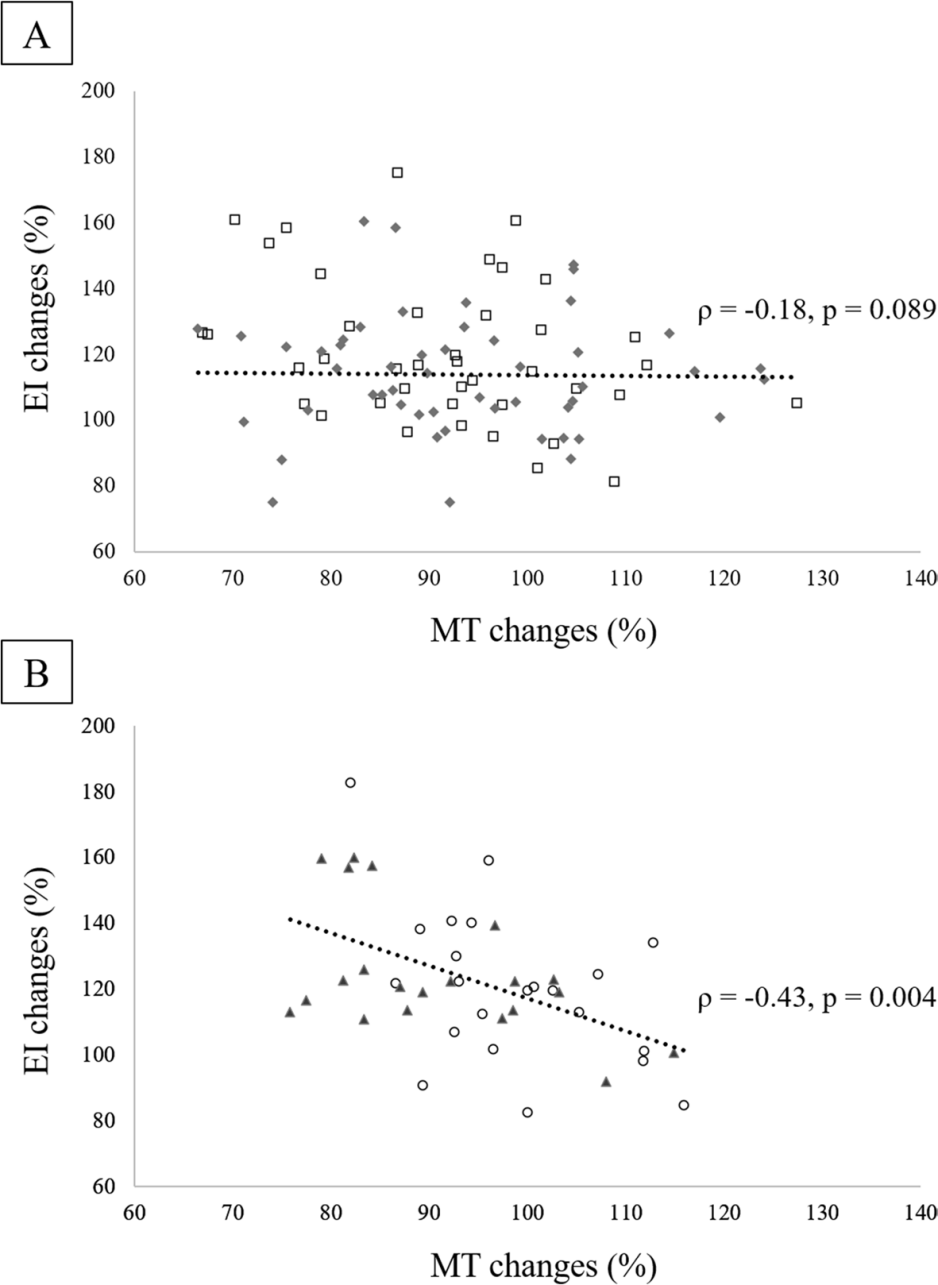
Scatter plot of the change ratios in MT and EI accompanied by the measurement postures. **A** Scatter plot for older. **B** Scatter plot for young. The vertical axis represents the change ratios in EI, and positive values mean that the EI is higher when sitting than when supine. The horizontal axis represents MT change ratios, and positive values mean that the MT is thicker when sitting than when supine. Each plot means older men (□), older women (◆), young men (〇), and young women (▲)

Table 3 shows the results of correlation analyses performed separately for the older and young subgroups. In both older and young participants, the correlation coefficients of MT and CSA and MV measured in the sitting posture were significantly higher than those in the supine posture. In young participants, the correlation coefficient between intraMAT and EI in the sitting posture was significantly higher than that in the supine posture (Supplemental Fig. 1a). In contrast, no significant difference was observed between the correlation coefficients in the older patients (Supplemental Fig. 1b). Furthermore, a significant negative correlation between the change ratios in MT and EI due to posture change was confirmed in young participants (*ρ* = −0.43, *p* = 0.004), but not in older participants (*ρ* = −0.18, *p* = 0.089; Fig. 4).

**Table 3:** Results of correlation coefficients and Meng’s test in the older and young subgroups

	Supine	Sitting	Meng’s test
	*r*-value	*r*-value	*p*-value
Between CSA and MT			
Older	**0.59**	**0.68**	< 0.001
Young	**0.69**	**0.75**	0.010
Between MV and MT			
Older	**0.59**	**0.72**	< 0.001
Young	**0.71**	**0.86**	< 0.001
Between intraMAT and EI			
Older	**0.51**	**0.49**	0.557
Young	**0.35**	**0.47**	0.002

The *r*-value indicates the correlation coefficient between MT and EI measured in the supine and sitting postures and CSA, MV, and intraMAT. The bold on *r*-values shows the statistical significance of the correlation coefficients. A *p*-value < 0.05 on Meng’s test indicates a significant difference between correlation coefficients amongst two measurement postures

## Discussion

The correlation coefficients between (a) MT and (b) CSA and MV were significantly higher when sitting than when supine, implying that MT measurements reflect VM muscle quantity more accurately when sitting. EI correlated significantly with intraMAT, and in young individuals, the correlation coefficient was significantly higher when sitting than when supine. The present study was the first to investigate posture effects on MT and EI measurements, and based on significantly stronger correlation coefficients, we recommend capturing US images of the VM in the sitting posture.

Giles et al. [25] reported that supine correlations of CSA and MV with MT were 0.73 and 0.65, respectively, in agreement with this study’s values of 0.65 (Fig. 3). Consistent with our hypothesis, MT-CSA and MT-MV correlations were significantly stronger when sitting than when supine. These results were also consistent in the older and young subgroups, and image acquisition in the sitting posture was superior to that in the supine posture for evaluating muscle quantity.

Posture change from the supine to sitting causes a decrease in the contact pressure between the posterior surfaces of the thigh and chair and an increase in gravity on the anterior thigh. These lead to changes in muscle shape, which is associated with a consequent decrease in MT when measured in sitting posture. During supine VM measurements, soft tissue, including muscle and subcutaneous fat, is compressed, and it can be difficult to adjust the probe to the maximum MT site because the MT site is not necessarily related to the observable femur location (Fig. 1[i]). In contrast, during sitting VM measurements, the MT location is directly in line with the femur apex, making it generally easier to identify the appropriate VM measurement site. Since easier site identification is expected to yield more consistent measurements, we hypothesized that sitting measurements would yield stronger correlations with MRI-based muscle quantity indices, and our results support this hypothesis. Although our results also showed smaller MT values in sitting vs. supine postures (Table 2), this is irrelevant to the ultimate goal of indicating muscle quantity; the indicator that has the maximum correlation with the target quantity should be preferentially used.

Experimental control of knee angle during measurement may be more important when sitting than when supine because presumably the natural range of sitting knee angles is larger than the natural range of supine knee angles. Generally, the muscle fascicle length increases along with the joint angle, resulting in a decrease of MT by the muscle stretched [26, 27]. That is, if one assumes that MT is directly affected by knee angle — because knee angle stretches and thus deforms the muscle — then knee angle control is necessary to achieve reliable measurements.

This study found no difference between the two measurement postures for the correlation between EI and intraMAT in all participants. A previous study [21] reported no difference in EI within the entire muscle length. In other words, muscle composition is unaffected by muscle morphology, stretching, and/or posture. However, our results indicated that the EI value in the sitting posture was higher than that in the supine posture (supine, 68.0 ± 15.4 a.u.; sitting, 78.1 ± 13.9 a.u. [Table 2]). In addition, the effect size of EI change between two measurement postures was relatively larger than that of MT change. This can be explained by the attenuation of the US beam according to depth; because the MT in the sitting posture was significantly thinner, depth-dependent attenuation of the US beam was likely reduced. In the young subgroup, the correlation coefficient between EI and intraMAT was higher in the sitting posture than in the supine posture (supine, *r* = 0.35; sitting, *r* = 0.47 [Table 3 and Supplemental Fig. 1a]). The VM in the young subgroup was thicker and influenced by greater US attenuation, especially in the supine posture. In contrast, thinning of MT in the sitting posture mitigates the attenuation effect. In fact, a decrease in MT due to postural changes from supine to sitting in young individuals was significantly associated with an increase in EI (refer to Fig. 4). Therefore, the correlation between EI and intraMAT became stronger in young individuals. These results suggest that the acquisition of US imaging in the sitting posture is more appropriate when sitting than when supine for MT and EI measurement in young individuals.

There were several limitations to the present study. First, only two postures, i.e. supine and sitting, were compared. VM measurements in the sitting posture included both effects of the pressure on the posterior distal thigh and muscle stretch of the VM due to knee flexion. Although the present study determined the effect of the pressure on the posterior distal thigh, the effect of muscle stretch on MT and EI measurements was not clear. Second, we could not exclude the presence of osteoarthritis (OA) because no radiographs were taken. Since OA is a common disease in the elderly, the participants in this study might have OA in one or more joints. Especially, decreased MT and enhanced EI are associated with functional disabilities and worse symptoms in patients with knee OA [28–30]. However, it is unclear whether sitting VM measurements are sufficient for detecting muscle degeneration of knee OA. Future studies are required to verify the efficacy of VM measurements in the sitting posture for knee OA patients.

## Conclusions

The results of this study indicated that MT was more closely associated with muscle quantity when sitting than when supine. Correlations between EI and intraMAT were significantly higher when sitting than when supine, especially in young individuals. These findings suggest that assessment of VM muscle quantity in the sitting posture is superior for young and older individuals, and assessment of VM muscle quality in the sitting posture is most effective for younger individuals with greater muscle quantity.

## Supplementary Information

**Additional file 1:** **Supplemental Fig. 1**. a Scatter plot for correlation analysis inyoung individuals. b Scatter plot for correlation analysis in older individuals.

## Data Availability

The datasets used and/or analysed during the current study are available from the corresponding author on reasonable request.

## Abbreviations

CSA: Cross-sectional area
EI: Echo intensity
IntraMAT: Intramuscular adipose tissue
ICC: Intraclass correlation coefficient
MRI: Magnetic resonance imaging
MT: Muscle thickness
MV: Muscle volume
OA: Osteoarthritis
ROI: Region of interest
TE: Echo time
TR: Repetition time
US: Ultrasound
VM: Vastus medialis
