# Supplementary material for: Sitting vs. supine ultrasound measurements of the vastus medialis: correlations with MRI measurements and age considerations
Source: J Physiol Anthropol. 2023 Jul 15;42:14. doi: 10.1186/s40101-023-00331-6 (PMC10350276; doi:10.1186/s40101-023-00331-6)
Supplement: Supplementary file 1 — Additional file 1: Supplemental Fig. 1. a Scatter plot for correlation analysis inyoung individuals. b Scatter plot for correlation analysis in older individuals. [file 40101_2023_331_MOESM1_ESM.docx]

**
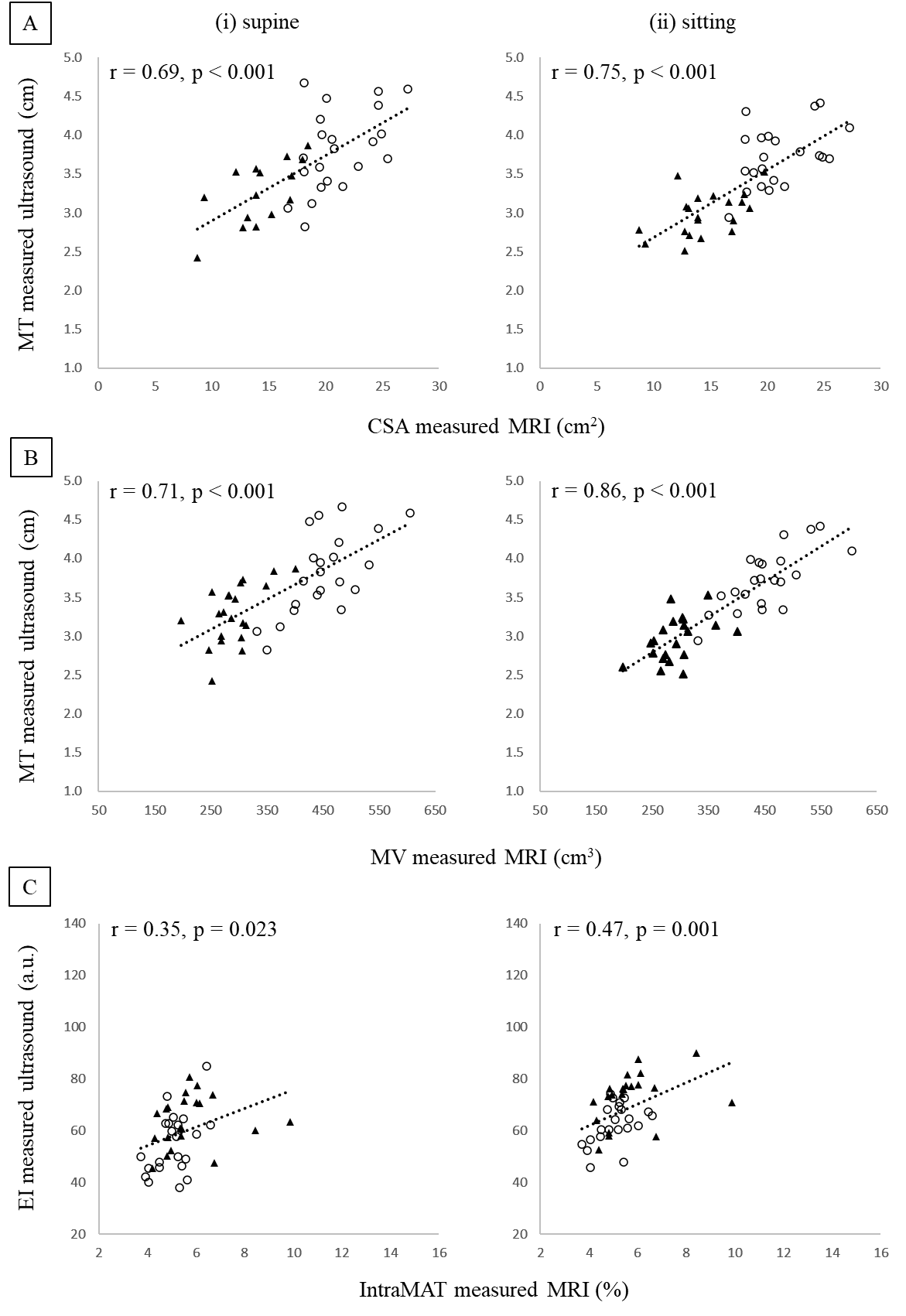
Supplemental Figures**

**Supplemental Figure 1a.** Scatter plot for correlation analysis in young individuals

**
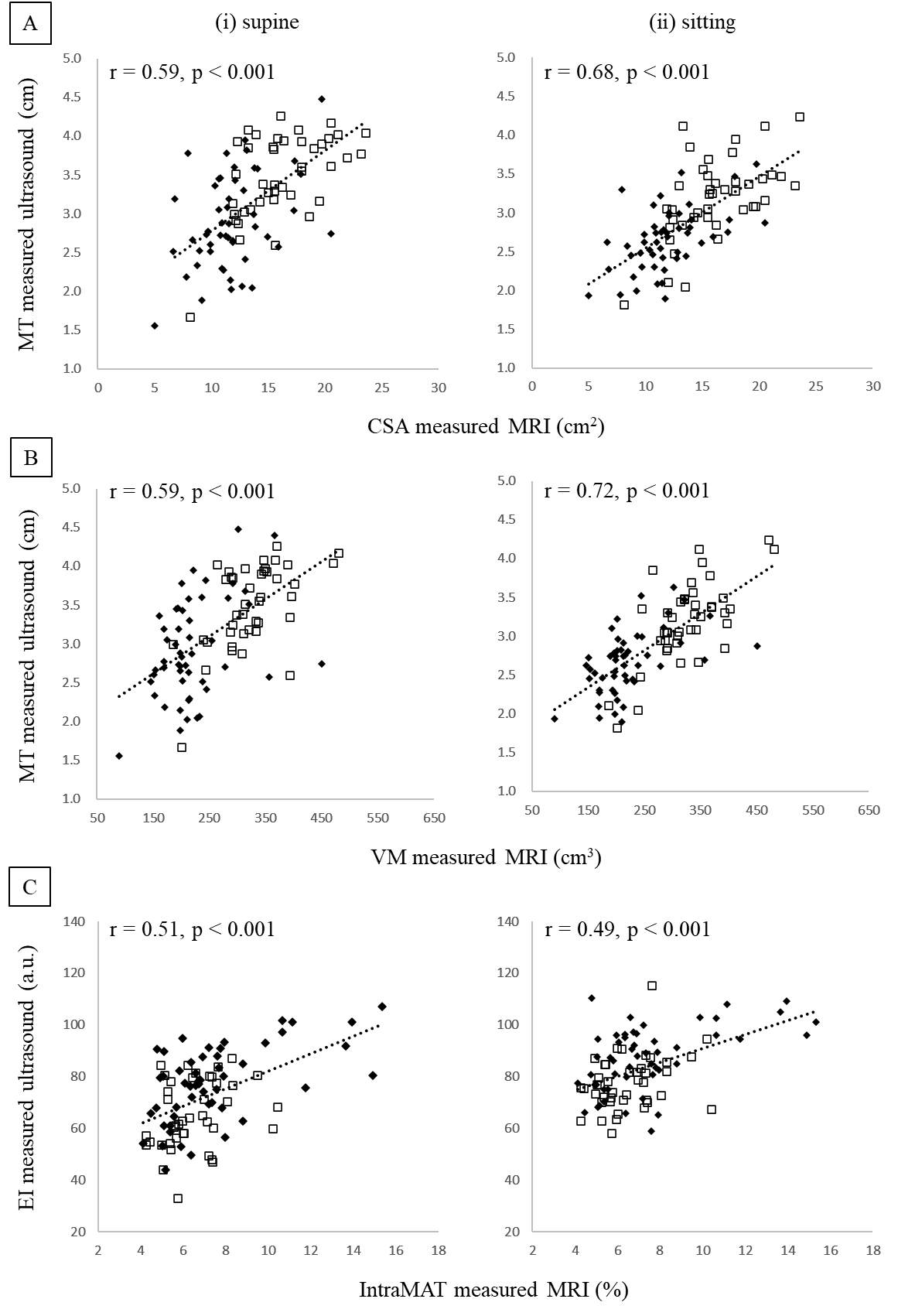
Supplemental Figure 1b.** Scatter plot for correlation analysis in older individuals

Caption:

A, Scatter plot of cross-sectional area and muscle thickness (MT) measured in (ⅰ) supine and (ⅱ) sitting postures

B, Scatter plot of muscle volume and MT measured in (ⅰ) supine and (ⅱ) sitting postures

C, Scatter plot of intramuscular adipose tissue and echo intensity measured in (ⅰ) supine and (ⅱ) sitting postures

A P-value < 0.05 (within each scatter plot) on the Pearson correlation coefﬁcient indicates a significant difference between parameters measured by magnetic resonance imaging and ultrasound.

Each plot means older men (□), older women (◆), young men (〇), and young women (▲).
